# Biological Characteristics and Pathogenicity of *Helcococcus ovis* Isolated From Clinical Bovine Mastitis in a Chinese Dairy Herd

**DOI:** 10.3389/fvets.2021.756438

**Published:** 2022-02-11

**Authors:** Kai Liu, Zhaoju Deng, Limei Zhang, Xiaolong Gu, Gang Liu, Yang Liu, Peng Chen, Jian Gao, Bo Han, Weijie Qu

**Affiliations:** ^1^College of Veterinary Medicine, Yunnan Agricultural University, Kunming, China; ^2^College of Veterinary Medicine, China Agricultural University, Beijing, China

**Keywords:** pathogenicity, phylogeny, antimicrobial resistance profile, *Helcococcus ovis*, clinical bovine mastitis

## Abstract

*Helcococcus ovis* (*H. ovis*) was first reported in ovine subclinical mastitis milk and post-mortem examination organs in Spain and the United Kingdom in 1999; subsequently, it appeared in cattle, horse, goat, and human. However, isolation and characterization of the pathogen from clinical bovine mastitis is unknown. The objective of this study was to identify the pathogen in clinical bovine mastitis. A total of four strains were isolated from bovine mastitis milk samples from a Chinese dairy farm, and they were identified as *H. ovis* by microscopic examination and 16S rRNA gene sequencing. Phylogenetic tree was constructed using 16S rRNA gene, and the isolates were closely related to other China strains and strains from Japan. The growth speed of the *H. ovis* isolated was relatively slower than *Streptococcus agalactiae*, and the phenotypic characteristics were similar to *H. ovis* CCUG37441 and CCUG39041 except to lactose. The isolates were sensitive to most of the common used antimicrobials. The *H. ovis* isolates could lead to mild murine mastitis alone and induce severe mastitis when co-infected with *Trueperella pyogenes* in the murine mammary infection model constructed.

## Introduction

Bovine mastitis is one of the most costly diseases in dairy industry due to milk discarding and related treatment costs, as well as culling of cows (1–3).

In China, the most frequently isolated mastitis pathogens were *Escherichia coli* (14.4%), *Klebsiella* spp. (13.0%), coagulase-negative staphylococci (11.3%), *Streptococcus dysgalactiae* (10.5%), and *Staphylococcus aureus* (10.2%) (4). Once these pathogens enter the mammary gland and detected by epithelial cells together with resident macrophages, the cells will release inflammatory cytokines such as TNF-α (tumor necrosis factor–alpha), IL-1β (interleukin-1 beta), humoral components (alexin, antibodies, antimicrobial peptides) are also participate in the process, and then, more leukocytes (neutrophils and lymphocytes) will be attracted under the chemotaxis, and more cytokines be synthesized and secreted; in the end, bovine mastitis is induced till the mastitis pathogens are eliminated (5).

Traditional diagnosis for bovine mastitis includes visual examination, California Mastitis Test, and Somatic Cells Counting. Pathogens isolation and identification are help for mastitis diagnosis and treatment; whereas, due to characteristics of microorganism, some of them are hard to isolate, which limits the research of such mastitis pathogens. Because of the limits of farm diagnostic tools in China, certain uncommon mastitis pathogens cannot be accurately identified on site. This might result in false negative results for mastitis diagnosis.

*Helcococcus ovis* (*H. ovis*) was first isolated from colonies mixed with *Trueperella pyogenes* (*T. pyogenes*) and *Staphylococcus* spp. from the lung, liver, spleen, and mastitis milk of two sheep in the United Kingdom and Spain (6), respectively. Subsequently, studies indicated that it can induce many other diseases in species of animals, even in human being, which suggest that it may be a zoonotic pathogen. *H. ovis* was also isolated from cows with abortions (7), puerperal metritis (8, 9), valvular endocarditis (10), horses with pulmonary abscess (11), and sheep with pleuritis and bronchopneumonia (12, 13).

Bacteriology methods and biochemistry tests are usually used to identify the biology characteristics of *H. ovis*; results indicated that this bacterium is a catalase-negative, facultatively anaerobic, and gram-positive cocci; and it often co-infected with other bacteria such as *Staphylococci* and *T. pyogenes*.

To the best of our knowledge, only four isolates of *H. ovis* were genome sequenced by Cunha et al. (8), whose results indicated that the guanine cytosine (GC) content of *H. ovis* was about 27.5%, the number of 33 tRNAs was identified in *H. ovis*, and all four isolates of *H. ovis* contained a ribosomal protection gene (*tetB*) and major facilitator superfamily (MFS) efflux gene (*tetA*), which confer resistance to tetracyclines. In addition, one of the isolates contained AcrEF-TolC, which can confer resistance to fluoroquinolones, cephalosporins, cephamycins, and penams (8).

Recently, *H. ovis* was found in human with pyogenic disease. The patient was reported to have a contact history with wool and cowhide leather before the disease and recovered after treatments of cefotaxime and ornidazole. The *H. ovis* was isolated from the samples collected from the skin around the eyes of the patient. Further antimicrobial resistance test indicated that the *H. ovis* strain is susceptible to penicillin, ampicillin, teicoplanin, ceftriaxone, vancomycin, and linezolid (14). Schwaiger et al. (15) reported coexistence of *H. ovis* with *T. pyogenes* detected by PCR; however, they failed to isolate the bacteria from the samples.

The objective of this study is to describe the *H. ovis* isolates first found in clinical mastitis cases in China, to determine the phylogeny relation to *H. ovis* strains isolated from other species, and investigate the antimicrobial resistance profiles and pathogenicity.

## Materials and Methods

### Statement of Ethics

All experiments followed the Regulations of Experimental Animals (2008) promulgated by China Ministry of Science and Technology. All animal procedures were approved by the Institutional Animal Care and Use Committee of Yunnan Agricultural University (Approval No: IACUC-20132030301).

### Isolation and Identification

During the daily etiology examination of clinical mastitis milk samples in a dairy farm in Hebei province, four blood agar plates with tiny and transparent colonies were classified as gram-positive *cocci* but could not be further classified into *Staphylococcus* spp. nor *Streptococcus* spp. by a Streptococcal grouping kit (Lancefield's classification kit, Hopebio, Qingdao, China) test.

To identify these isolates, the four blood agar plates were delivered to the Mastitis Diagnostic Laboratory in China Agriculture University (Beijing). The pin-point colonies were transplanted onto blood agar plates and were incubated in 37°C until 36 h. Heavy growth of two types of colonies was observed, one was tiny and transparent, whereas the other type was white. All the two types of colonies were exposed to gram-staining and microscope examination.

Partial 16S rRNA gene (1,300 bp) amplification (forward primer: 5′-TACCTTGTTACGACTT-3′; reverse primer: 5′-AGAGTTTGATCCTGGCTCAG-3′) (16) and sequencing were conducted, and these sequences were BLAST with the available sequences in GenBank. The phylogenetic tree of these *H. ovis* isolates was constructed using the clustal V method (DNASTAR Lasergene-Megalign, version 7.1).

### Growth Curve of *H. ovis*

The growth curves of *H. ovis* and *S. agalactiae* were assessed simultaneously as *S. agalactiae* is one of the typical G^+^ cocci pathogens of bovine mastitis. The volume of 1 ml of culture media of each isolate was incubated into 100-ml brain heart infusion broth with 5% fetal bovine serum and placed on a constant temperature shaker (37°C, 220 rpm). The volume of 3 ml of bacterial suspension was collected at 0, 2, 4, 6, 8, 10, 12, 14, 16, 18, 20, 22, and 24 h, respectively, for optical density (OD) examination at 600 nm in a UV spectrophotometer (Jingke Scientific Instrument Co., Ltd., Shanghai, China).

### Biochemistry Test

Biochemical tests were performed on the four isolates using an HBI biochemical kit according to the manufacturer's instructions (Hopebio, Qingdao, China). Isolates were plated onto tryptic soy blood agar, and then, single colony was suspended into saline equivalent to a turbidity of 0.5 McFarland standard. The volume of 200 μl of saline suspension was added into each micro biochemical pool and then incubated at 37°C for 24 h. The culture medium will turn yellow when the test is positive to ribose, mannitol, sorbitol, lactose, raffinose, maltose, melibiose, and sucrose; red to urease; modena to hippurate hydrolysis; and blue to esterase.

### Antimicrobial Resistance Test

Antimicrobial resistance tests were performed using the broth microdilution method with *Streptococcus pneumoniae* ATCC 49619 as quality control strain according to the Clinical and Laboratory Standards Institute (17). Common used antimicrobials for bovine mastitis treatment and in human medicine was selected for antimicrobial resistance testing, which includes penicillin, cefalexin, ceftiofur, oxacillin, clindamycin, tetracycline, enrofloxacin, daptomycin, erythromycin, and vancomycin (18). The dosage was based on previous research (14). Plates were incubated at 37°C in a humidified atmosphere for 24 h. The minimum inhibitory concentration (MIC) of each strain was defined as the lowest concentration of an antimicrobial that completely inhibited growth in broth (no growth) during the 24-h incubation. The MIC was determined as per the CLSI's guidelines. The concentrations of the antimicrobials agents range from 0.015 to 16 μg/ml. Isolates were classified following the clinical breakpoints described in CLSI (17).

### Murine Mammary Infection Model of *H. ovis*

Pregnant (20 days of gestation) 6- to 8-week-old SPF BALB/c mice (SiPeiFu Laboratory Animal Technology, Beijing, China) were used to determine the pathogenic role of *H. ovis* during intramammary infection, as described before (19). On the third day after parturition, mice were anesthetized with intramuscular injection of Zoletil 50 (50 mg/kg; Virbac, Carros, France). Four groups (*n* = 5 per group) of mice were allocated with three challenge groups (12, 24, and 36 h) and one negative control group [sterile phosphate buffered saline (PBS)]. Teat ducts of both the L4 (left) and R4 (right) abdominal mammary glands were exposed under a binocular stereoscopic microscope, and 100 μl of bacterial suspension (10^5^ CFU/ml) was injected using a syringe with a 34G blunt needle (19). The clinical signs and pathological changes of each mouse were observed and recorded. Then, at 12, 24, and 36 h after challenge (five mice for each time point), the murine mammary gland was separated, part of mammary gland tissue (0.1 g) was separated into sterile tube under a germ-free environment to measure the bacterial burden, and the other part of mammary gland tissue was separated and fixed in 5% of paraformaldehyde to conduct histological evaluation.

### Murine Mammary Co-infection Model *H. ovis* and *T. pyogenes*

To illustrate the pathogenicity of the co-infection of *H. ovis* and *T. pyogenes*, four groups (*n* = 5 per group) of the abovementioned mice were allocated with three challenge groups (*H. ovis, T. pyogenes*, and *H. ovis* and *T. pyogenes*) and one negative control group (sterile PBS). The challenge method and dosage is same (10^5^ CFU/ml) with the single infection model above. The clinical signs and pathological changes of each mouse were observed and recorded. Murine blood was collected for serum separation to conduct ELISA at 24 h after challenge. Then, at 24 h after challenge (five mice for each time point), histological evaluation and immunohistochemical assay were conducted, as in the single challenge model above.

### ELISA

The IL-16 in blood was measured by ELISA kits (MULTI SCIENCES, Hangzhou, Jiangsu, China) according to the manufacturer's instructions. The absorbance was read at 450 nm by Multiskan MK3 (Thermo-Fisher Scientific, Waltham, MA, USA). All absorbance results were normalized by standard curves.

### Histological Analysis and Immunohistochemical Assay

After embedded in paraffin wax, and sectioned and stained with hematoxylin-eosin, histological evaluation was performed to assess tissue necrosis, polymorphonuclear neutrophilic granulocyte inflammation (i.e., neutrophilic inflammation), and lymphocytic inflammation. For immunohistochemical assays, the sections were incubated with rabbit immunoglobulin G (IgG) against mouse IL-1β or TNF-α and then with a HRP-conjugated secondary antibody. A microscope (Olympus, Tokyo, Japan) was used for the examination.

### Statistical Analysis

The prevalence of *H. ovis* and its 95% confidence interval (95% CI) was calculated by using online tool VassarStats (http://www.vassarstats.net/).

All experiments (growth curve determination, bacterial burden, and ELISA) consisted of three independent repeats, and results were analyzed using SPSS 20 and GraphPad Prism 8. 0. 2. Data were assessed using one-way analysis of variance (one- way ANOVA). The data were expressed as the mean ± SD. *P*-values < 0.05 were significant.

## Results

### Bacteria Isolation and Identification

**Figure 1:**
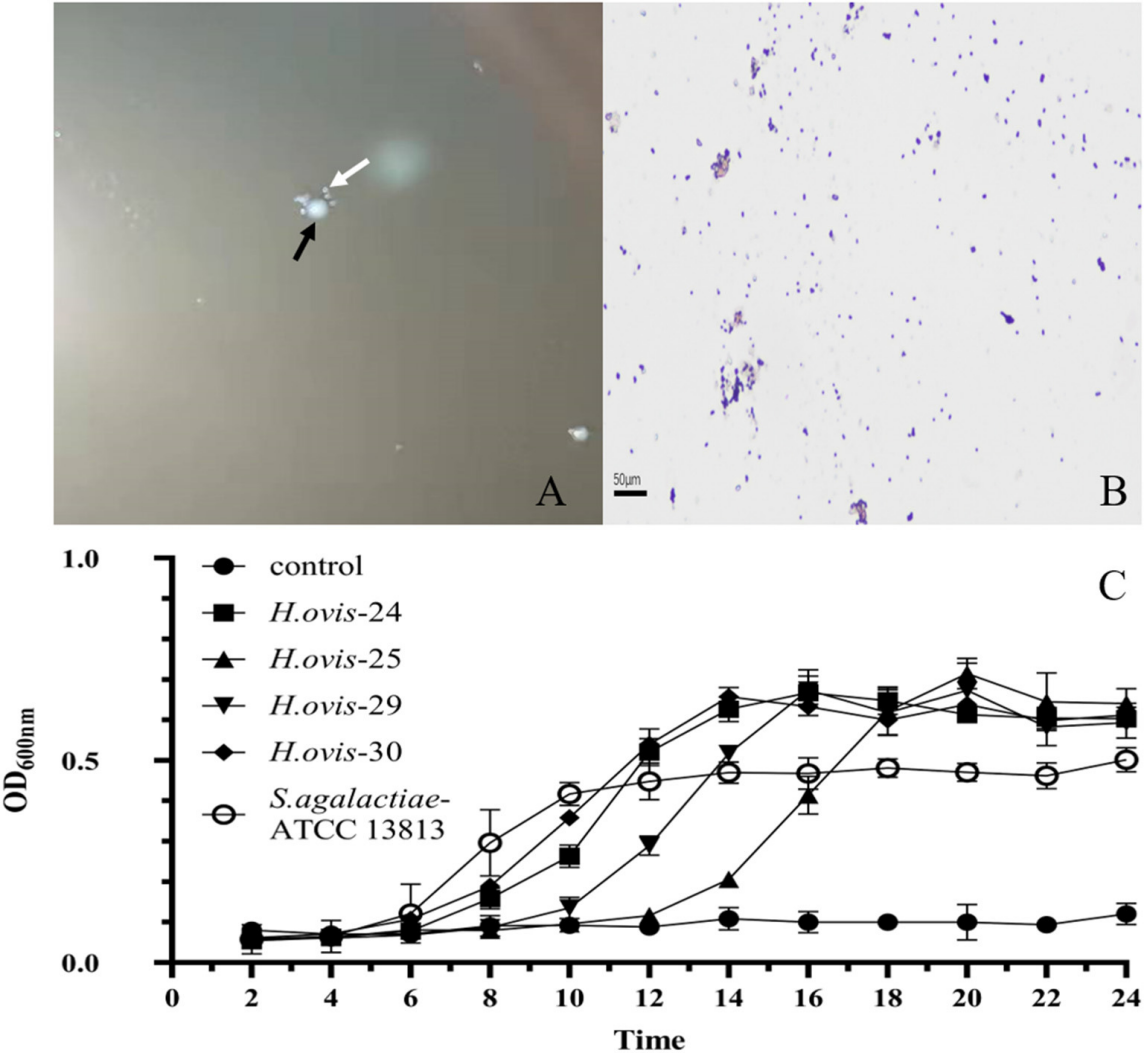
Morphological characteristics and growth curves of *Helcococcus ovis*. **(A)** White arrow shows the tiny, transparent colony; the black arrow indicates white colony. **(B)** Gram-positive cocci (400×). **(C)** Gram-positive irregularity rod-shaped bacteria (400×).

**Figure 2:**
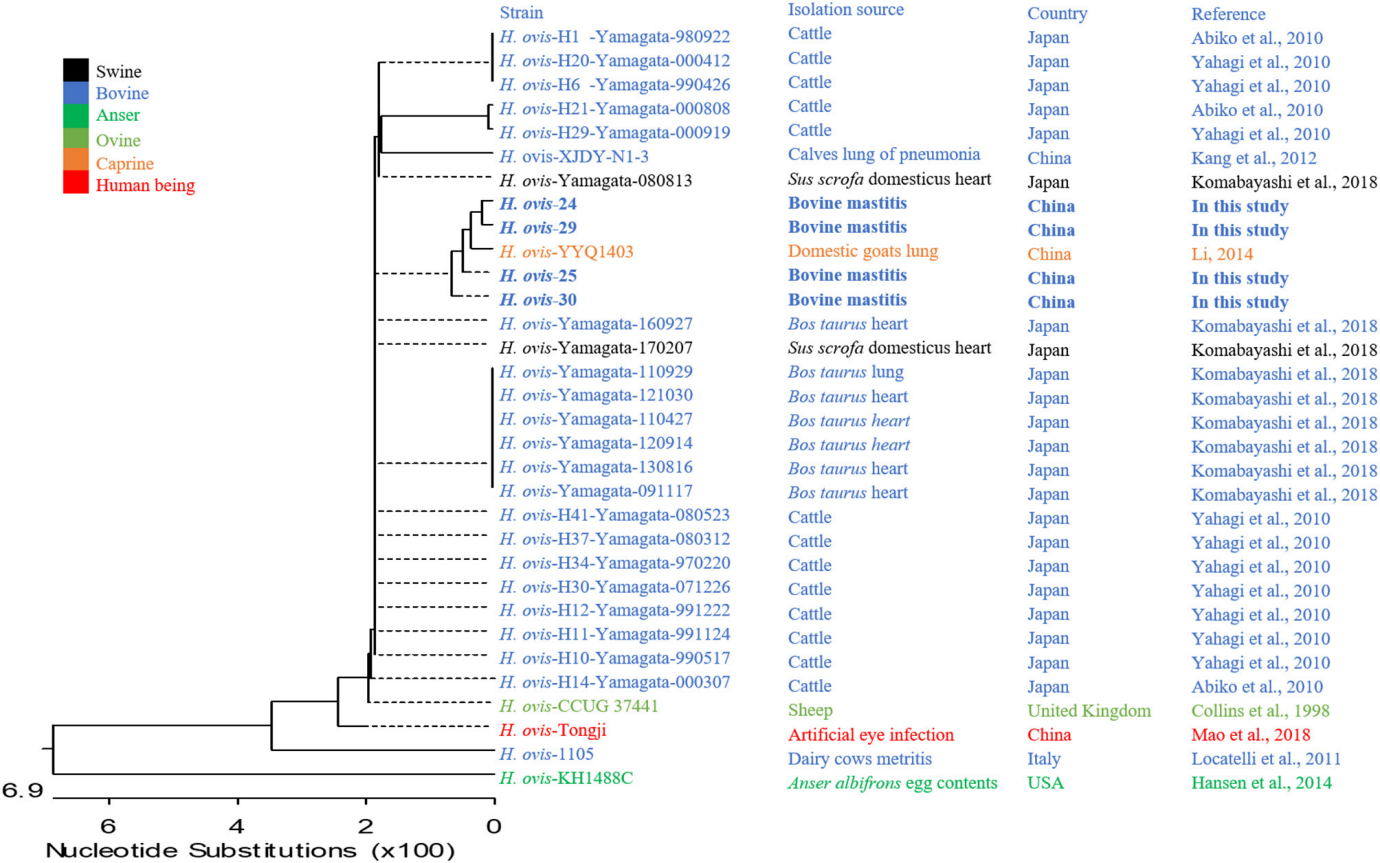
Phylogenetic analysis of four strains of *Helcococcus ovis*. Bold fonts indicate *Helcococcus ovis* isolates in this study.

The small and transparent colonies were single or in pair gram-positive *cocci*, whereas the white colonies were gram-positive single irregular rod-shaped bacteria (Figure 1). The gram-positive *cocci* were identified as *H. ovis*, whereas the rod-shaped bacteria were identified as *T. pyogenes* [information of the four *H. ovis* isolates (prevalence: 11.76%, 95% CI: 4.67–26.62%) was shown in Table 1]. The sequences of the *H. ovis* isolates were submitted to GenBank (https://www.ncbi.nlm.nih.gov/genbank/) with the accession numbers MT758192.1, MT758194.1, MT758195.1, and MT758196.1. All the four isolates (bold fonts) were closely related to the strains isolated from goat (*H. ovis*–YYQ1403) and bovine case (*H. ovis*–XJDY-N1-3) in China; meanwhile, they were also closely related to strains isolated from swine and bovine cases in Japan (e.g., *H. ovis*–Ymagata-080813 and *H. ovis*–Ymagata-160927) (Figure 2).

**Table 1:** Information of four isolates of *Helcococcus ovis*.

**Place**	**Isolate identity**	**Quarter**	**Mastitis grade (20)**	**Date**	**Pathogen**
Hebei	24	Left rear	II	July 31, 2019	*H. ovis, T. pyogenes*
Hebei	25	Left front	III	July 31, 2019	*H. ovis, T. pyogenes*
Hebei	29	Left rear	II	July 31, 2019	*H. ovis, T. pyogenes*
Hebei	30	Left front	I	July 31, 2019	*H. ovis, T. pyogenes*

### Growth Curve of *H. ovis*

The growth curve of the 4 *H. ovis* isolates consisted of a lag phase (~6 h), a log phase (~10 h), and a stationary phase. The *H. ovis* isolates have a long lag phase and relatively higher OD_600nm_ value comparing with *Streptococcus agalactiae* (Figure 1D).

### Biochemistry Test

The ribose, mannitol, sorbitol, raffinose, melibiose, sucrose, β-mannosidase, urease, hippurate, hydrolysis, and esterase reactions were negative for the four isolates while positive to lactose and maltose (Table 2). The biochemistry test characterization of the *H. ovis* isolates was same to the standard strain of *H. ovis* CCUG 37441 and CCUG 39041 except for lactose and esterase.

**Table 2:** Biochemical characteristics of the four *Helcococcus ovis* isolates.

**Phenotypic characteristic**	**Reported** ***H. ovis*** **(****10****)**		***H. ovis*** **in this study**			
	**CCUG37441**	**CCUG39041**	**24**	**25**	**29**	**30**
Ribose	**–**	**–**	**–**	**–**	**–**	**–**
Mannitol	**–**	**–**	**–**	**–**	**–**	**–**
Sorbitol	**–**	**–**	**–**	**–**	**–**	**–**
Lactose	**–**	**–**	**+**	**+**	**+**	**+**
Raffinose	**–**	**–**	**–**	**–**	**–**	**–**
Maltose	**+**	**+**	**+**	**+**	**+**	**+**
Melibiose	**–**	**–**	**–**	**–**	**–**	**–**
Sucrose	**–**	**–**	**–**	**–**	**–**	**–**
β-Mannosidase	**–**	**–**	**–**	**–**	**–**	**–**
Urease	**–**	**–**	**–**	**–**	**–**	**–**
Hippurate hydrolysis	**–**	**–**	**–**	**–**	**–**	**–**
Esterase	**+**	**+**	**–**	**–**	**–**	**–**

### Antimicrobial Resistance Test

As shown in Table 3, results showed that all isolates were susceptible to penicillin, cefalexin, ceftiofur, oxacillin, clindamycin, erythromycin, and vancomycin; except, one isolate (Hebei-24) was intermediate to tetracycline and enrofloxacin.

**Table 3:** Antimicrobial resistance profiles of the four *Helcococcus ovis* isolates.

**Antimicrobials**	**Isolate MIC (μg ml** ^**−1**^ **)**				**Breakpoints (μg ml** ^**−1**^ **)**		
	**Hebei-24**	**Hebei-25**	**Hebei-29**	**Hebei-30**	**S**	**I**	**R**
Penicillin	0.015	0.015	0.015	0.015	≤ 0.12	—	≥0.25
Cefalexin	1.000	1.000	1.000	0.500	—	—	≥8
Ceftiofur	<0.015	<0.015	<0.015	<0.015	—	—	≥8
Oxacillin	0.015	<0.015	<0.015	<0.015	≤ 2	—	≥4
Clindamycin	0.250	0.250	0.250	0.250	≤ 0.5	1-2	≥4
Tetracycline	8.000	0.500	0.500	0.500	≤ 4	8	≥16
Enrofloxacin	4.000	0.500	1.000	0.500	≤ 2	4	≥8
Daptomycin	0.25	0.015	0.25	0.25	≤ 1	—	—
Erythromycin	<0.015	<0.015	<0.015	<0.015	≤ 0.5	1-4	≥8
Vancomycin	0.125	0.125	0.250	0.125	≤ 2	4-8	≥16

*S, sensitive; I, intermediate; R, resistant. Breakpoints refer to Staphylococcus aureus (17) that were used as (14) demonstrated: penicillin ≥ 0.25μg ml^−1^; cefalexin ≥ 8μg ml^−1^; oxacillin ≥ 4μg ml^−1^; clindamycin ≥ 4μg ml^−1^; tetracycline ≥ 16μg ml^−1^; enrofloxacin ≥ 8μg ml^−1^; daptomycin ≥ 1μg ml^−1^; erythromycin ≥ 8μg ml^−1^; and vancomycin ≥ 16μg ml^−1^. Unshaded cells indicate that the MIC value was sensitive, light gray shading indicates intermediate*.

### Inflammation of Murine Mammary Gland Infected by *H. ovis*

**Figure 3:**
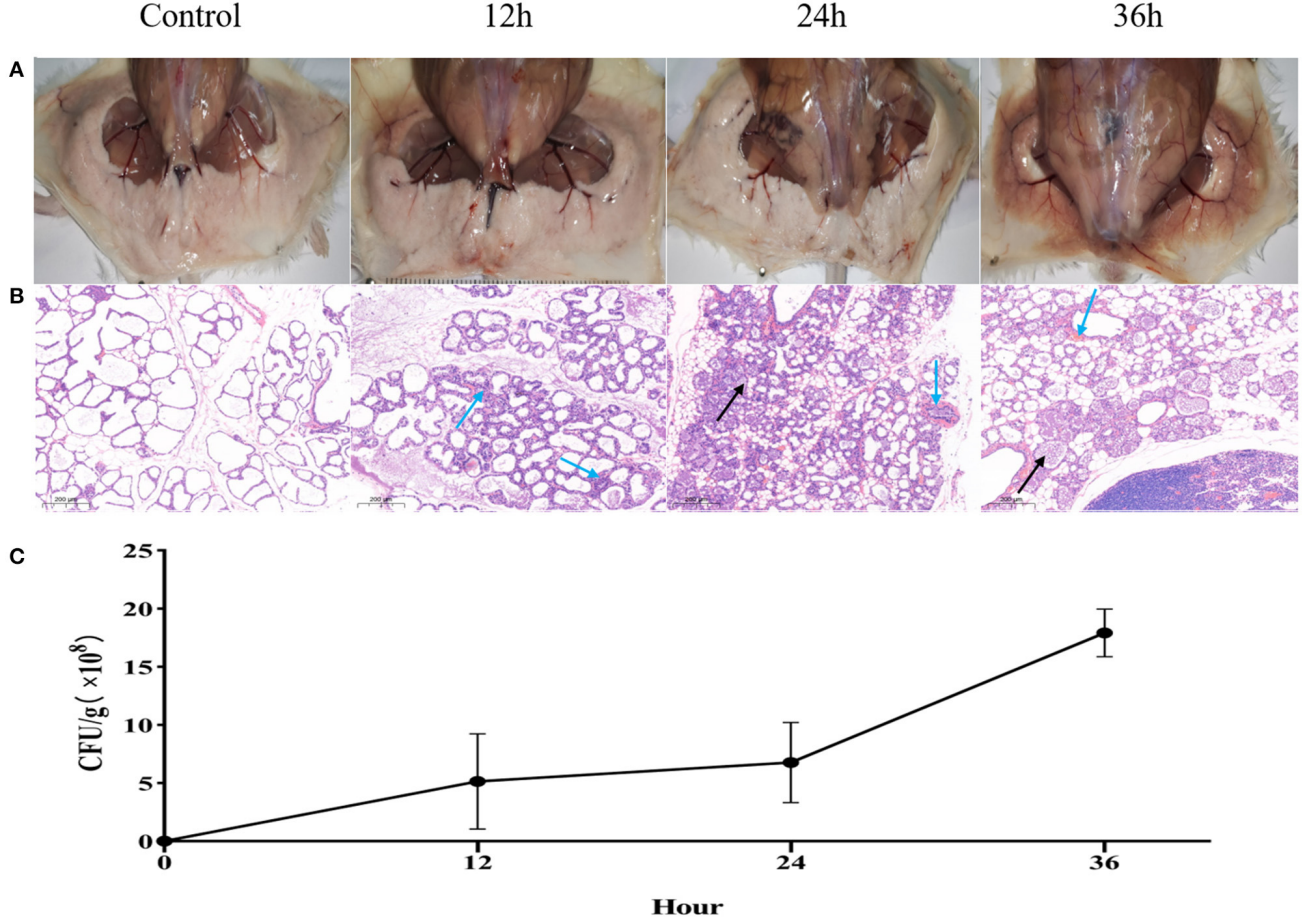
Inflammation and histological evaluation and bacterial burden of murine mammary gland after intramammary inoculation with *Helcococcus ovis*. **(A)** Pathological changes in murine mammary glands challenged with *Helcococcus ovis*. **(B)** *Helcococcus ovis* infection induced slight murine mastitis: infiltration of inflammatory cells (mainly neutrophils) into gland alveoli (black arrow) and stromal hyperplasia and progressive mammary alveolar damage (blue arrow). **(C)** Bacterial burden in mammary glands of mice (up to 36 h after inoculation). Each time point represents five mice in each of the four groups. Data are mean ± SD of three independent experiments.

Swollen and hyperemia mammary glands were observed 12 h after challenge with *H. ovis*, and more profound pathological changes observed after 24 and 36 h (Figure 3A) after challenge. For histological characteristics of murine mammary glands (see Figure 3B). Slight infiltrations of inflammatory cells and progressive mammary alveolar damage were observed, stromal hyperplasia appeared in the infected mammary gland 12 and 24 h after challenge, large quantity of lymphocytes formed a tumor-like structure after 36 h, and degeneration and necrosis of mammary epithelial cells were observed. The bacterial load of the *H. ovis* isolates in the murine mammary gland tissue was 4.6 × 10^8^ CFU/g after 12-h challenge and gradually increased gradually to 6.8 × 10^8^ CFU/g and 1.8 × 10^9^ CFU/g at 24 and 36 h after challenge (Figure 3C).

### Pathogenicity of Co-infection of *H. ovis* and *T. pyogenes*

**Figure 4:**
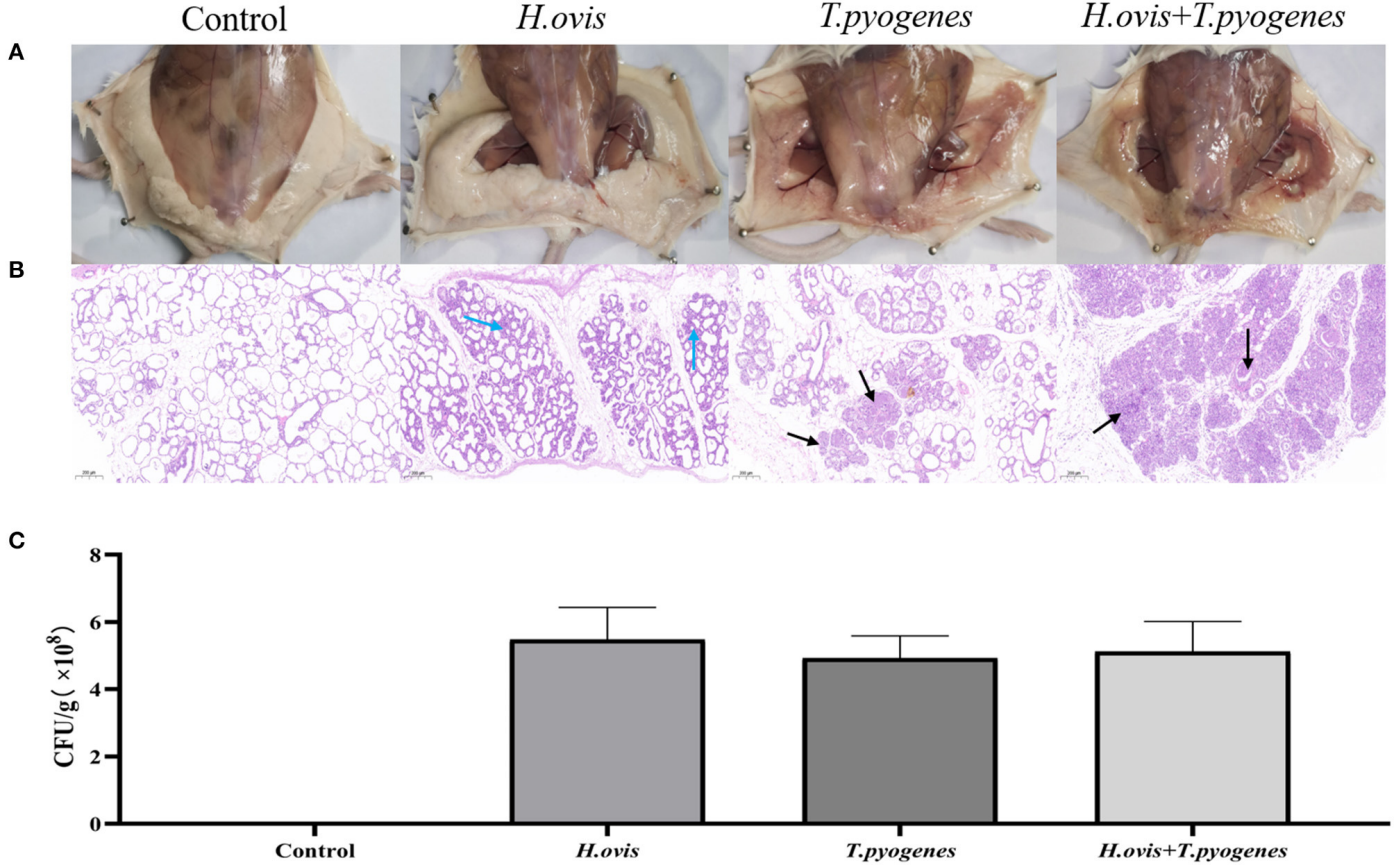
Inflammation and histological evaluation and bacterial burden of murine mammary gland after intramammary inoculation with *Helcococcus ovis, Trueperella pyogenes, Helcococcus ovis*, and *Trueperella pyogenes*. **(A)** Pathological changes in murine mammary glands challenged with *Helcococcus ovis, Trueperella pyogenes, Helcococcus ovis*, and *Trueperella pyogenes*. **(B)** *Helcococcus ovis* induced murine mastitis, with stromal hyperplasia and mammary alveolar damage (blue arrow) after infection. *Trueperella pyogenes* induced murine mastitis, with an increased infiltration of inflammatory cells (mainly neutrophils) into the gland alveoli (black arrow) and progressive mammary alveolar damage after infection. *Helcococcus ovis* and *Trueperella pyogenes* co-infection induced murine mastitis, with an increasing number of inflammatory cells (mainly neutrophils) infiltrated into the gland alveoli (black arrow), stromal hyperplasia, and progressive mammary alveolar damage after infection. **(C)** Bacterial burden in mammary glands of mice (24 h after inoculation). Each group represents five mice in each of the four groups. Data are mean ± SD of three independent experiments.

At 24-h after challenge, the bacterial load was even among the three infected groups (Figure 4C). Swollen, hyperemia, and edema mammary gland were observed after individual challenge with *H. ovis* and *T. pyogenes* separately; severer pathological changes exhibited in the group co-infected by *H. ovis* and *T. pyogenes* (Figure 4A). Histological examination revealed severer inflammation in the co-infection group than the individual challenge group: stromal hyperplasia, slight infiltrations of inflammatory cells, and mammary alveolar damage exhibited in *H. ovis* infection group; numerous inflammatory cells infiltrations and mammary alveolar damage were observed in *T. pyogenes* infection group; whereas massive infiltrations of inflammatory cells and stromal hyperplasia together with mammary alveolar damage exhibited in the *H. ovis* and *T. pyogenes* co-infection group (Figure 4B).

**Figure 5:**
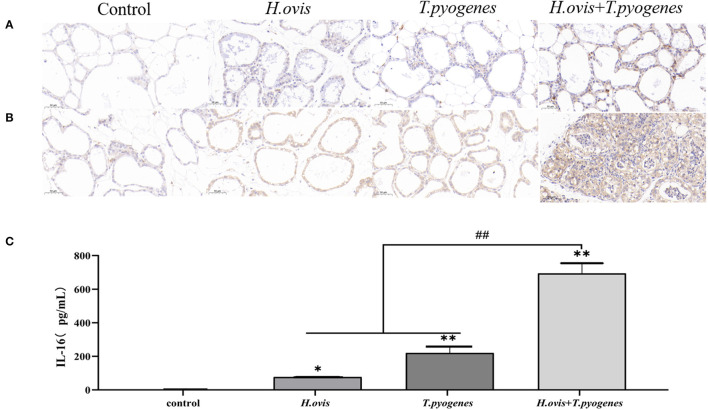
Immunohistochemical (IHC) detection of IL-1β and TNF-α in murine mammary glands and enzyme linked immunosorbent assay (ELISA) detection of IL-16 in murine serum after intramammary inoculation with *Helcococcus ovis, Trueperella pyogenes, Helcococcus ovis*, and *Trueperella pyogenes*. **(A)** IHC detection of IL-1β in murine mammary glands challenged with *Helcococcus ovis, Trueperella pyogenes, Helcococcus ovis*, and *Trueperella pyogenes*. **(B)** IHC detection of TNF-α in murine mammary glands challenged with *Helcococcus ovis, Trueperella pyogenes, Helcococcus ovis*, and *Trueperella pyogenes*. **(C)** ELISA detection of IL-16 in murine serum challenged with *Helcococcus ovis, Trueperella pyogenes, Helcococcus ovis*, and *Trueperella pyogenes*. Data are mean ± SD of three independent experiments. ^*^P < 0.05, ^**^ and *##P* < 0.01.

The intensity of brown color revealed that TNF-α and IL-1β in murine mammary gland was upregulated in the co-infection group than the *H. ovis* and *T. pyogenes* individual infectiongroups in immunohistochemical analysis. The expression of IL-16 in murine serum was increased significantly in the co-infection group than in the individual infection group by the ELISA test results (Figure 5).

## Discussion

To the best of our knowledge, this study is the first report of *H. ovis* isolated from bovine mastitis in China, although the prevalence was relatively low. In China, there were only four publications of *H. ovis*, which includes three cases in goat and bovine pneumonia (18, 21, 22) and one in human artificial eye, which raised concern over the zoonotic property of *H. ovis* (14). More mastitis milk samples should be invesgated and etiological research need to be conducted to confirm the pathogenicity of *H. ovis* in bovine mastitis and other infection diseases according to Koch's postulates. Rothschild (11) assumed that this bacteria is one of the skin microbiota of cows, which also required further study. The colony of *H. ovis* is so tiny that it may be ignored by laboratory technicians when mixes with other major bacteria or milk droplets during routine milk sample examination. Similar with mycoplasma, it is a critical mastitis pathogen but difficult to isolate due to slow growth and tiny colonies, which makes it hard to diagnose and research (23). Molecular diagnostics can overcome this and, at the same time, etiology investigation of an emerging pathogen is also pivotal.

Bovine mastitis is predominantly caused by bacteria; therefore, antimicrobials are extensively applied to mastitis prevention and treatment (19), which raised the concern of AMR that threatens human health (18). In this study, the resistance of the four isolates to daptomycin was observed, and one of the isolates was intermediary resistant to tetracycline and enrofloxacin, which were common used in treatments against bovine mastitis. Genome sequencing of *H. ovis* isolated from bovine puerperal metritis in previous research has indicated that the isolates in that study contained tetracycline-resistant gene: a ribosomal protection gene (*tetB*) and MFS efflux gene (*tetA*), and one of the isolates contained AcrEF-TolC, an inner membrane proton that confers resistance to fluoroquinolones, cephalosporins, cephamycins, and penams (8). However, in our study, only slight resistance to antimicrobials was demonstrated, which may indicated that the isolates were rarely exposed to antimicrobial treatments and their emergence in mastitis milk was recent.

The phenotypic characteristics difference of the four isolates with CCUG 37441 and CCUG 39041 may be due to the fact that isolates were isolated from mastitis milk and that lactose was the main carbohydrate source. Because of the unreliability of biochemical methods, they can only be used as auxiliary inspection method, and molecular identification is required to confirm it (14).

The murine model of intramammary challenge with bovine mastitis pathogens has been successfully used to assess bacterial infection and tissue damage (19). The murine model of *H. ovis* may improve our understanding on the correlation between this bacteria and bovine mastitis, the treatment efficacy applied in clinical trials, as well as the relationship among the bacteria, bovine immune response, and lactation (24). The mammary epithelial cells and the resident macrophages are the first line to interact with pathogens at the onset of mastitis, and then, cytophagocytosis is activated, and pro-inflammatory cytokines such as TNF-α, IL-1β, and IL-8 are released. Consequently, more immunocytes such as neutrophils and lymphocytes are gathered to the inflammation site, and then, more inflammatory cytokines secreted and inflammatory worsened (5).

In this study, the pathogenicity of *H. ovis* to mice was examnied at different periods after challenge. The slight infiltrations of inflammatory cells, stromal hyperplasia, mild mammary alveolar damage, and bacteria burden in tissue indicated that *H. ovis* cannot cause severe mastitis. Disease caused by the co-infection of *H. ovis* and other pathogens have been reported before (6, 9, 10), whereas the pathogenicity of the co-infection *H. ovis* infection has not been researched. To discover the pathogenicity of the co-infection of *H. ovis* and *T. pyogenes*, an advanced study was conducted. Individual infection by *T. pyogenes* leads to typical bovine mastitis (such as the symptoms of redness, swelling, heat, pain, and dysfunction of mammary glands, as well as massive infiltration of inflammatory cells, and mammary alveolar damage) in this study, which was consistent with previous study that *T. pyogenes* was distributed in variaty of animals and can lead to bovine mastitis (25). Although the co-infection of *H. ovis* and *T. pyogenes* induced severer mastitis than individual infections by each of the two bacteria, the murine mammary gland alveolar was filled with rupt epithelial cells and neutrophils. Leukocytes are always circulating in the blood around mammary gland; when the barriers of mammary gland are broke through by bacteria, the epithelial cells are damaged, cytokines such as IL-1β and TNF-α are released, and chemotaxis moves neutrophils into alveolar to eliminate the invading pathogen. When the infection occurs and neutrophils apoptosis being delayed, it helps limiting the extent of infection (5). The infection of *H. ovis* and *T. pyogenes* also increased the IL-16 concentration in murine serum. As IL-16 is secreted by T cells and is chemoattractant to CD4^+^T cells, the two bacteria may be able to activate cellular immunity. The interaction among microbiota plays a crucial role in biofilm forming, metabolism, and pathogenicity (26–29). Stipkovits also demonstrated the synergy between *Mycoplasma arginini* (*M. arginini*) and *Streptococcus dysgalactiae* (*Strep. dysgalactiae*); due to the ability of *M. arginini* to inhibit the T cells growth and the cytotoxic T cells activity, *M. arginini* infection does not produce clinical signs of mastitis but it can induce severe mastitis together with *Strep. dysgalactiae* (30). In this study, *H. ovis* was isolated from samples that *T. pyogenes* exist, and the co-infection of *H. ovis and T. pyogenes* caused much serious murine mastitis than individual infection by *T. pyogenes*; meanwhile, the challenge with *H. ovis* alone only caused mild mastitis, which means that *H. ovis* may be able to induce immunosuppression in cows or improve the inflammation.

## Conclusions

The *H. ovis* isolates in our research were closely related to other strains isolated from China and the strains from Japan, the growth speed of the isolates was relatively slower than *S. agalactiae*, and the phenotypic characteristics were similar to CCUG37441 and CCUG39041 except to lactose; isolates were sensitive to most of antimicrobials; *H. ovis* could lead to mild murine mastitis and could induce severe mastitis when co-infected with *T. pyogenes*. As an emerging pathogen, it draws importance when designing mastitis control plans against *H. ovis*.

## Data Availability Statement

The datasets presented in this study can be found in online repositories. The names of the repository/repositories and accession number(s) can be found in the article/supplementary material.

## Ethics Statement

The animal study was reviewed and approved by Institutional Animal Care and Use Committee of Yunnan Agricultural University (Approval No: IACUC-20132030301).

## Author Contributions

The study was designed by KL, LZ, and XG. The experiments were performed by YL and PC. The data were analyzed by JG, WQ, and BH, who also drafted the manuscript, which was reviewed and revised by ZD and GL. The final version was read and approved by all authors.

## Funding

This study was supported by the National Natural Science Foundation of China (no. 31660730), Yunnan Provincial Science and Technology Department-Yunnan Expert Workstation (no. 202005AF150041), and Science Research Foundation of Yunnan Education Bureau (grant no. 2020y134).

## Conflict of Interest

The authors declare that the research was conducted in the absence of any commercial or financial relationships that could be construed as a potential conflict of interest.

## Publisher's Note

All claims expressed in this article are solely those of the authors and do not necessarily represent those of their affiliated organizations, or those of the publisher, the editors and the reviewers. Any product that may be evaluated in this article, or claim that may be made by its manufacturer, is not guaranteed or endorsed by the publisher.
